# Near chromosome–level genome assembly of the microsporidium *Hamiltosporidium tvaerminnensis*

**DOI:** 10.1093/g3journal/jkad185

**Published:** 2023-08-11

**Authors:** Pascal Angst, Jean-François Pombert, Dieter Ebert, Peter D Fields

**Affiliations:** Department of Environmental Sciences, Zoology, University of Basel, Basel 4051, Switzerland; Department of Biology, Illinois Institute of Technology, Chicago, IL 60616, USA; Department of Environmental Sciences, Zoology, University of Basel, Basel 4051, Switzerland; Department of Environmental Sciences, Zoology, University of Basel, Basel 4051, Switzerland

**Keywords:** fungi, microsporidia, telomere-to-telomere, Iso-Seq, alternative polyadenylation, splicing efficiency, *Hamiltosporidium tvaerminnensis*

## Abstract

Microsporidia are intracellular parasitic fungi whose genomes rank among the smallest of all known eukaryotes. A number of outstanding questions remain concerning the evolution of their large-scale variation in genome architecture, responsible for genome size variation of more than an order of magnitude. This genome report presents the first near-chromosomal assembly of a large-genome microsporidium, *Hamiltosporidium tvaerminnensis*. Combined Oxford Nanopore, Pacific Biosciences (PacBio), and Illumina sequencing led to a genome assembly of 17 contigs, 11 of which represent complete chromosomes. Our assembly is 21.64 Mb in length, has an N50 of 1.44 Mb, and consists of 39.56% interspersed repeats. We introduce a novel approach in microsporidia, PacBio Iso-Seq, as part of a larger annotation pipeline for obtaining high-quality annotations of 3,573 protein-coding genes. Based on direct evidence from the full-length Iso-Seq transcripts, we present evidence for alternative polyadenylation and variation in splicing efficiency, which are potential regulation mechanisms for gene expression in microsporidia. The generated high-quality genome assembly is a necessary resource for comparative genomics that will help elucidate the evolution of genome architecture in response to intracellular parasitism.

**Significance Statement**

Microsporidia are a model for genome evolution in response to intracellular parasitism, but we lack high-quality resources from species with large genomes. We present a near-complete assembly of a large-genome microsporidium, *Hamiltosporidium tvaerminnensis*, and obtain high-quality gene annotations through full-length transcripts using Iso-Seq, a novel approach in microsporidia. Our study provides insights into gene regulation and paves the way for comparative genomic analyses aiming to understand the evolution of genome reduction and expansion in these intracellular parasites.

## Introduction

Microsporidia have become a valuable model in evolutionary biology for studying extreme parasitism (Murareanu *et al*. 2021; Wadi and Reinke 2020). Compared to their fungi relatives, the genomes of microsporidia have been strongly shaped by their specialized life history of obligate intracellular parasitism (Corradi 2015). In particular, the genomes of most microsporidia are very compact and reduced (Murareanu *et al*. 2021). However, the genome sizes of microsporidia vary by more than an order of magnitude, making them a suitable model for studying genome evolution in eukaryotes and parasites (Wadi and Reinke 2020). A powerful approach for this is comparative genomics (i.e. comparing the presence or absence of genetic sequences, their relative location, and their abundance among different species), for which high-quality genome assemblies are a prerequisite (Kille *et al*. 2022). Increasing interest in microsporidia has boosted available genome resources, but most published genomes are drafts (Williams *et al*. 2022). Following recent successful efforts to obtain telomere-to-telomere assemblies in *Encephalitozoon* (Mascarenhas dos Santos *et al*. 2023), a genus of microsporidia with small genomes, we focus here on a large-genome species, *Hamiltosporidium tvaerminnensis*.

First noted by Green (1957) as *Octosporea bayeri*, the reclassified *H. tvaerminnensis* has become a model in the study of host–parasite interactions with its only host *Daphnia magna*, a planktonic microcrustacean (Altermatt *et al*. 2007; Lass and Ebert 2005; Orlansky and Ben-Ami 2019; Routtu and Ebert 2015; Santos and Ebert 2022; Vizoso and Ebert 2004). Haag *et al*. (2011) provided the cytological and molecular description of *H. tvaerminnensis*, and Corradi *et al*. (2009) produced the first draft of its genome. Recently, genetic and genomic studies have started using *H. tvaerminnensis* to investigate the evolution of variation in microsporidian genome architecture (Angst *et al*. 2022, 2023; de Albuquerque *et al*. 2020; Haag *et al*. 2013, 2020). *H. tvaerminnensis* has a remarkably large genome compared to other microsporidia, partly due to the proliferation of transposable genomic elements (de Albuquerque *et al*. 2020; Parisot *et al*. 2014). Draft genomes are available for the nearest known relative, *Hamiltosporidium magnivora* (Haag *et al*. 2020), and more distantly related species, *Thelohania contejeani* (Cormier, Wattier, *et al*. 2021) and *Edhazardia aedis* (Desjardins *et al*. 2015).

Demands for comparative genomic approaches are increasing, calling for an improved genome of *H. tvaerminnensis*, a leading model organism among large-genome microsporidia. A high-quality genome will allow advancements in the study of genome architecture evolution in microsporidia, for example, through comparison with chromosomal assemblies of *Encephalitozoon*. We have integrated Pacific Biosciences (PacBio) Continuous Long Reads (CLR), Oxford Nanopore Technologies (ONT), and Illumina DNA sequencing for a near-chromosomal assembly, and we employed PacBio Iso-Seq for high-quality gene annotation. The first use of Iso-Seq for a microsporidium provides insights into microsporidian alternative polyadenylation (APA) and splicing efficiency as potential regulation mechanisms of gene expression in reduced genomes.

## Materials and methods

### Sample preparation and sequencing

The *H. tvaerminnensis* isolate used in this study was obtained from the *D. magna* genotype FI-OER-3-3, collected in July 2005 from a rockpool (59°47′18.9″N, 23°10′26.9″E) on the Oeren Island in the Tvaerminne archipelago, Southwestern Finland. The same isolate has been sequenced with Illumina short reads for earlier genome drafts (Corradi *et al*. 2009; Haag *et al*. 2020) and a biogeographic study (Angst *et al*. 2022). Whole-genome shotgun sequencing with Illumina short reads from the latter was reused here (NCBI database, SRA accession: SRX13146514, Bioproject ID: PRJNA780787). From the *D. magna* genotype FI-OER-3-3 infected with *H. tvaerminnensis* at different stages of its life cycle, we obtained host and microsporidia tissue free from other microbiota by treating host individuals with 3 antibiotics (streptomycin, tetracycline, and ampicillin), each at a concentration of 50 mg/L, for 3 days and by feeding them with Sephadex G-25 Superfine (GE Healthcare, Chicago, IL, USA) at a concentration of 5 g/L following Fields *et al*. (2015). We extracted high-molecular–weight DNA from these individuals using Genomic-tips (QIAGEN, Hilden, Germany) for generating long-read sequencing data. A standard PacBio (Menlo Park, CA, USA) genomic DNA library and sequencing on a SMRT Cell in CLR mode on a Sequel I has been done by the Quantitative Genomics Facility service platform at the Department of Biosystem Science and Engineering (D-BSSE, ETH) in Basel, Switzerland. Additionally, an ONT (Oxford, UK) genomic DNA library was prepared with the SQK-LSK110 ligation kit and sequenced on a MinION device with a Spot-ON Flow Cell (R9.4.1).

To extract full-length transcripts, we used the RNeasy extraction kit (QIAGEN) supplying Proteinase K for protein digestion. The NEBNext Single Cell/Low Input cDNA Synthesis and Amplification Module kit (New England Biolabs, Ipswich, MA, USA) was used to generate cDNA from the extraction. An Iso-Seq cDNA library was generated with the Iso-Seq Express Oligo Kit and the SMRTbell Express Template Prep Kit 2.0 (PacBio). The Iso-Seq library was sequenced using a single SMRT Cell on a Sequel I at the D-BSSE (Basel, Switzerland) and processed with the *isoseq3* pipeline (https://github.com/PacificBiosciences/IsoSeq), resulting in ∼8.5 Gb of circular consensus sequence (CCS) data.

### Genome assembly

An assembly based on the PacBio CLR sequencing data, generated with Canu v.2.1.1 (Koren *et al*. 2017), was extended in silico using a seed-based approach with Consed v.29 (Gordon *et al*. 1998) as described in Pombert *et al*. (2013). Nonmicrosporidia sequences have been identified and removed from this assembly using the BlobToolKit v.2 (Challis *et al*. 2020) and by mapping Illumina data from an uninfected host genotype (Angst *et al*. 2022). Independently, the Nanopore sequencing reads were basecalled with bonito v.0.5.3 (https://github.com/nanoporetech/bonito) using the dna_r9.4.1_e8.1_sup@v3.3 model and then assembled with NextDenovo v.2.5.0 (https://github.com/Nextomics/NextDenovo) using an expected genome size of 20 Mb. The Nanopore NextDenovo assembly was polished with the Illumina reads and the PacBio CLR reads using NextPolish v.1.4.1 (Hu *et al*. 2020), Medaka v.1.7.2 (https://github.com/nanoporetech/medaka), and 2 runs of Pilon v.1.24 (Walker *et al*. 2014) and then merged with the extended PacBio CLR assembly using quickmerge v.0.3 (Solares *et al*. 2018) and manual curation. The absence of contaminants in this final assembly was ascertained by BLAST homology searches against the NCBI database, and its completeness was assessed with check_for_telomeres.pl v.0.3 (Mascarenhas dos Santos *et al*. 2023). Repeats in the assembly were detected with RepeatModeler v.2.0.2, including the LTR pipeline (Flynn *et al*. 2020) and ReapeatMasker v.4.1.2 (Smit *et al*. 2013) and plotted with karyoploteR v.1.24.0 (Gel and Serra 2017) in R v.4.2.2 (R Core Team 2022).

### Genome annotation

The *H. tvaerminnensis* genome was annotated by feeding the Iso-Seq transcriptome data as extrinsic evidence to the fungal annotation pipeline funannotate v.1.8.13 (Palmer and Stajich 2022). Briefly, as per the guideline for gene prediction using long RNA reads (https://github.com/Gaius-Augustus/BRAKER/blob/master/docs/long_reads/long_read_protocol.md), we mapped the whole-transcript reads from the CCS FASTA produced by *isoseq3* to the repeat-masked genome using Minimap2 v.2.22 (Li, 2018) with the flags “-ax splice –secondary=no -C5”. Approximately 25.5% of the reads aligned to the parasite genome. We then collapsed the total read set into distinct transcripts (while retaining isoforms) using the *collapse_isoforms_by_sam.py* script provided by the *cDNA_Cupcake* (https://github.com/Magdoll/cDNA_Cupcake) pipeline and ran GeneMarkS-T v.5.1 (Tang *et al*. 2015) to predict protein-coding regions in the transcripts. The resultant FASTA and GFF files were used for gene prediction and annotation with the wrapper tool funannotate, which relies on TANTAN v.26 (Frith 2011) for repeat masking. Funannotate operated AUGUSTUS v.3.4.0 (Stanke *et al*. 2006, 2008), GeneMark-ES v.4.62 (Brůna *et al*. 2020), GlimmerHMM v.3.0.4 (Majoros *et al*. 2004), SNAP v.2013_11_29 (Korf 2004), and EVidenceModeler v.1.1.1 (Haas *et al*. 2008) for ab initio gene prediction and consensus gene structure generation. Functional annotation in funannotate relies on the combined evidence from InterProScan v.5.55_88.0 (Jones *et al*. 2014), eggNOG-mapper v.2.1.9 (Cantalapiedra *et al*. 2021; Huerta-Cepas *et al*. 2019), Phobius v.1.01 (Käll *et al*. 2004), SignalP v.5.0b (Almagro Armenteros *et al*. 2019), and antiSMASH v.6.0 (Blin *et al*. 2021) as well as on comparisons to Pfam (Mistry *et al*. 2021), UniProt (The UniProt Consortium 2023), MEROPS (Rawlings *et al*. 2014), dbCAN (Yin *et al*. 2012), and BUSCO v.5.4.3 (Manni *et al*. 2021) databases. Also, the completeness was assessed using BUSCO with its microsporidia_odb10 database (creation date: 2020-08-05), which encompasses 600 genes. For proteins without functional annotation from this pipeline, we lifted over functional annotations from the species reference proteome (UniProt database, Proteome ID: UP000292282) using BLAST v.2.3.0+ (Camacho *et al*. 2009), AGAT v.1.0.0 (Dainat 2022), and GFF3sort v.1.0.0 (Zhu *et al*. 2017).

### Full-length transcript analyses

For the analysis of splicing efficiency and APA, we followed the methodology described in Schärfen *et al*. (2022). Briefly, starting from the BAM file, which was provided by the sequencing facility, we used lima v.2.6.0 (https://github.com/PacificBiosciences/barcoding) in “-isoseq” mode to remove Iso-Seq template-switching oligo sequences and to orient subreads in the correct 5′ to 3′ direction. After aligning subreads to our genome using Minimap2 in “splice:hq” mode, we used SAMTools v.1.16.1 (Li *et al*. 2009) and BEDTools v.2.30.0 (Quinlan 2014) to identify intron-spanning reads. The percentage of spliced introns was calculated by dividing the number of spliced reads by the total number of reads for each intron times 100. For this analysis, introns were manually selected based on Iso-Seq coverage level. R was used to test potential associations between the percent of spliced reads, intron length, and whether introns are in frame or not with Spearman's correlation and a Wilcoxon test, respectively.

For the analysis of APA, briefly, we used SAMTools, BEDTools, and pysam v.0.20.0 (https://github.com/pysam-developers/pysam) to extract each read's 3′-end coordinate following Schärfen *et al*. (2022). Then, we drew the distribution of 3′-end coordinates as a histogram separately for each gene and applied the find_peaks algorithm from scipy.signal v.1.7.3 (Virtanen *et al*. 2020) with a height equal to 4 and a distance equal to 18. Hence, we found the number of peaks (= 3′-end coordinates) per gene. We then separately extracted the sequences of the 500 most expressed genes up to 40 nucleotides upstream of the highest peak, the second highest peak, and the third highest peak using seqtk v.1.3-r106 (https://github.com/lh3/seqtk). For each set of sequences, we did a motif analysis using XSTREME v5.5.0 from the MEME Suite (Grant and Bailey 2021) with a minimum motif length of 4 and determined the distance of the obtained motif from the peak.

## Results and discussion

### Genome sequencing and assembly

**Fig. 1.**
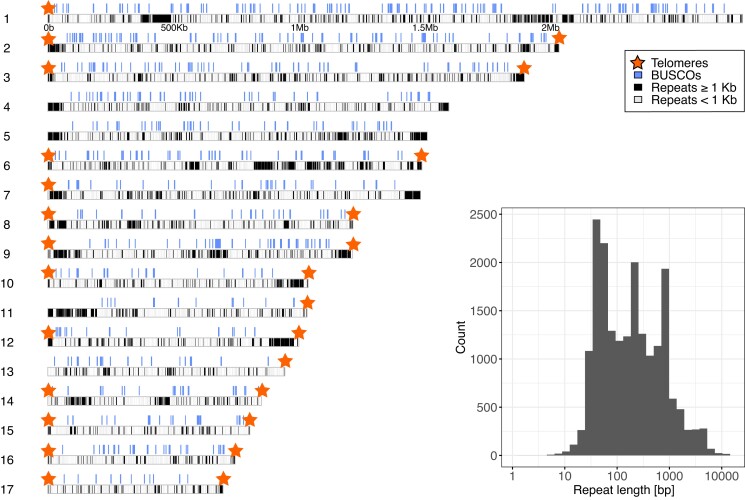
Karyoplot of *H. tvaerminnensis*. Eleven of the 17 contigs have 2 telomeres, i.e. represent full chromosomes. In total, 39.56% of the genome is composed of interspersed repeats and low complexity DNA sequences. The inset shows the repeat length distribution on a base-10 logarithmic scale.

We generated a genome assembly of *H. tvaerminnensis* based on PacBio CLR and ONT sequencing reads (total/N50: 17.53 Gb/1.54 Kb and 2.75 Gb/32.68 Kb, respectively). In a subsequent step, we polished this assembly using Illumina paired-end sequencing reads (total: 66 Gb). The final assembly consisted of 21.64 Mb in 17 contigs with an N50 of 1.44 Mb (Table 1; Fig. 1). We used BUSCO to assess the biological completeness of our assembly. We found a score, as compared to the microsporidia BUSCO reference set, of 94%, a relatively high value for a large-genome microsporidium (Cormier, Chebbi, *et al*. 2021). The structural completeness of our assembly was assessed using scripts from Mascarenhas dos Santos *et al*. (2023), which allow for the identification of telomeres. Telomeres are identifiable by terminal, short, tandem repeats–(TTAGGG)*_n_* in the case of *H. tvaerminnensis*, as is the case in most fungi (Rahnama *et al*. 2020). Eleven contigs represented complete chromosomes flanked by telomeres at both ends (Fig. 1; Supplementary Table 1), 4 contigs had only 1 telomere, and 2 had none. Therefore, we estimated that the genome contains 13–17 chromosomes. Large multicopy repeats arrayed in tandem and scattered throughout the genome prevented us from fully assembling the chromosomes with major repetitive loci abutting contig ends without telomeres (Fig. 1). Overall, 39.56% of the genome consisted of interspersed repeats (retroelements 7.07%, DNA transposons 7.04%, other 25.45%) with a mean length of 477 bp and a maximum length of 17.12 Kb (Fig. 1). High numbers of repeats are common in large-genome microsporidia (de Albuquerque *et al*. 2020), explaining why previous attempts to assemble the genome of *H. tvaerminnensis* have been less successful in both completeness and contiguity (size = 18.34 Mb; number of contigs = 2,915; N50 = 9.58 Kb; BUSCO score = 87.6%) (Haag *et al*. 2020) compared to our long-read sequencing-based assembly. The highest quality genomes within the microsporidia are from the genus *Encephalitozoon*, which have an order of magnitude smaller genomes than *H*. *tvaerminnensis* and telomeric repeats of (TTAGG)*_n_* on both ends of all eleven chromosomes (Mascarenhas dos Santos *et al*. 2023).

**Table 1.** Statistics of the assembly and annotation of the *H. tvaerminnensis* genome.

Assembly		Annotation	
Total length (Mb)	21.64	Total length of repeats (Mb)	8.56
GC content (%)	26.60	Number of protein-coding genes	3,573
Contig N50 (Mb)	1.44	Mean gene length (bp)	1,391
Contig number	17	Number of predicted introns	98
BUSCO completeness score (%)	94	Number of Iso-Seq confirmed introns	17
		Number of genes with confirmed introns	15
		Mean intron length (bp)	28

### Iso-Seq for genome annotation

RNA-seq data from intracellular parasites are sparse, and even more sparse are full-length transcript sequences. Iso-Seq helped us predict both gene structures and UTRs more accurately than was previously done for microsporidia. Using 8.5 Gb of Iso-Seq reads (so-called CCS of cDNA), we obtained annotations for 3,573 protein-coding genes with a mean length of 1,391 bp (Table 1). The number of genes in microsporidia ranges from about 2,000 to 4,000 and is positively correlated with genome size (Jespersen *et al*. 2022). Compared to previous work, we obtained fewer fragmented genes; the score of fragmented BUSCOs in our annotations was 1.8% (previously 3.8%; Haag *et al*. 2020). We also obtained many fewer introns (98) than previously predicted (890). However, our number is still higher than estimates for other large-genome microsporidia annotated with the help of (short-read) RNA-seq (Desjardins *et al*. 2015). After observing the discrepancy between annotations with and without RNA-seq data, we suspected introns may not be efficiently spliced.

### Splicing efficiency analysis

Species with reduced genomes, sparse introns or no introns, and reduced spliceosomes have been suggested to exhibit low splicing efficiencies (Schärfen *et al*. 2022; Whelan *et al*. 2019). Microsporidia are such species. For example, in *Encephalitozoon cuniculi*, splicing efficiency for small introns is only around 20% (Grisdale *et al*. 2013). We used direct evidence from full-length transcripts and found high variation in splicing efficiency in *H. tvaerminnensis* as has previously been reported for the reduced genomes of the red alga *Cyanidioschyzon merolae* (Schärfen *et al*. 2022) and for parasitic fungi (Sieber *et al*. 2018). From the 98 predicted introns, we used 17 introns from 15 genes with good representation in terms of sequencing coverage in the Iso-Seq data (Supplementary Table 2). These introns had a mean length of 28 bp. The percentages of spliced transcripts ranged from 7.5 to 92.3% (Supplementary Table 2), with a mean of 54.53%. We found neither a statistical correlation between the percent spliced and the length of introns nor an association between the percent spliced and whether introns were in frame or not. In conclusion, we found fewer introns in *H. tvaerminnensis* than previously suggested and intermediate splicing efficiencies, which align with theorized patterns for reduced-genome species. All but 2 introns identified in *H. tvaerminnensis* featured the same splice sites as have been found in (non-)parasitic fungi (i.e. 5′GU…AG3′) (Kupfer *et al*. 2004). The other 2 introns had splice sites 5′AA…AG3′ and 5′AU…GA3′.

### APA

The mechanisms of gene regulation in microsporidia are unexplored. APA is a widespread and conserved regulatory mechanism by which the availability of multiple polyadenylation sites can lead to variation in 3′-UTR lengths of mRNA isoforms and thus 3′-UTR content, which strongly influences gene expression (Mitschka and Mayr 2022). Here, Iso-Seq data allowed us to investigate the occurrence of mRNA isoforms encoding the same protein but differing in their 3′-UTRs resulting from APA. By observing multiple 3′-ends for many genes, we found direct evidence for APA in the full-length transcripts of *H. tvaerminnensis*. Upstream of the 3′-ends, a motif analysis of the 500 most expressed genes revealed enrichment of a UAAA tetramer. The UAAA tetramer was found in 95.8% of the genes, with 87.7% of them part of an A[A/U]UAAA hexamer on average 20 nucleotides upstream of the most common 3′-ends. A[A/U]UAAA is a known motif for cleavage of protein-coding mRNAs in metazoa (Neve *et al*. 2017) and is shown to be enriched upstream of 3′-ends in the bee-infecting microsporidium *Nosema ceranae* (Chen *et al*. 2021). These motifs were slightly less enriched upstream of the second and third most common 3′-ends (Supplementary Table 3). No motif was strongly enriched that did not contain an UAAA tetramer.

## Supplementary Material

jkad185_Supplementary_Data

## Data Availability

Raw data are deposited in the NCBI SRA database, and the assembled genome plus the predicted protein sequences are available in the NCBI GenBank database (FIOER33 v3; BioProject ID PRJNA778105) and at https://doi.org/10.6084/m9.figshare.22794887. Analysis scripts are available at https://github.com/pascalangst/Angst_etal_2023_G3. Supplemental material available at G3 online.
